# Upregulation of Tim‐3 is associated with poor prognosis in acute myeloid leukemia

**DOI:** 10.1002/cam4.5549

**Published:** 2022-12-21

**Authors:** Zhengwei Wu, Jiawang Ou, Nannan Liu, Zhixiang Wang, Junjie Chen, Zihong Cai, Xiaoli Liu, Xiao Yu, Min Dai, Hongsheng Zhou

**Affiliations:** ^1^ Department of Hematology, Nanfang Hospital Southern Medical University Guangzhou China; ^2^ Department of Immunology, School of Basic Medical Sciences Southern Medical University Guangzhou China

**Keywords:** acute myeloid leukemia, immune response, leukemia stem cell, prognosis, T‐cell immunoglobulin mucin 3

## Abstract

Acute myeloid leukemia (AML) is a heterogeneous hematopoietic malignancy originated from leukemia stem cells (LSC). Emerging evidence suggests T‐cell immunoglobulin mucin‐3(Tim3) as surface marker for LSC. However, the clinical significance and biology of Tim‐3 in AML remain to be determined, especially those LSCs. In public AML databases as well as our data, we separated AML patients into Tim‐3^high^ and Tim‐3^low^ subsets using the X‐tile software and evaluated the associations between Tim‐3 and overall survival (OS) and disease‐free survival (DFS). The Cancer Genome Atlas (TCGA) cohort revealed that high Tim‐3 expression in leukemic cells was linked with poor prognosis (DFS: *p* = 0.018; OS: *p* = 0.041). Furthermore, multiple regression analysis shows that Tim‐3 was an independent factor for the prognosis (HR = 2.26, 95% CI = 1.15–4.44, *p* = 0.017). Validation cohort of public gene expression omnibus (GEO) confirmed that Tim‐3 was a prognostic candidate in AML. Besides, in our internal cohort, we also confirmed that over expression of Tim‐3 protein in LSC/LPC made poor prognosis in AML. Additionally, we revealed that the LSC markers *AKR1C3*, *CD34,* and *MMRN1* were upregulated in the Tim‐3^high^ group of TCGA. We found that the upregulated genes in the Tim‐3^high^ group were mainly enriched in immune response, cytokine binding and cell adhesion molecules, and JAK–STAT signaling pathway, by gene ontology (GO) enrichment analysis and Kyoto Encyclopedia of Genes and Genomes (KEGG) analysis. Collectively, we revealed that, for the first time, upregulation of Tim‐3 in LSCs at the level of gene and protein expression is associated with poor prognosis and the important biological feature of Tim‐3 of LSC in AML.

## Graphical abstract

Up‐regulation of Tim‐3 in LSCs at the level of gene and protein expression is associated with poor prognosis and the important biological feature of Tim‐3 of LSC in AML.;Up regulated genes in Tim‐3high group were mainly enriched in immune response, cytokine binding and cell adhesion molecules, and JAK‐STAT signaling pathway, by gene ontology (GO) enrichment analysis and Kyoto Encyclopedia of Genes and Genomes (KEGG) analysis.

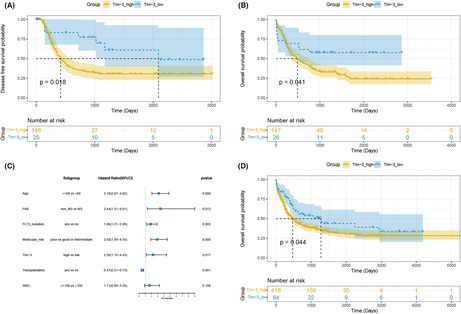

## 1 INTRODUCTION

Acute myeloid leukemia (AML) is a kind of hematological malignancy originating from hematopoietic stem/progenitor cells, presenting heterogeneous clinical manifestations and prognosis. Labeling the special markers of leukemia cells, especially leukemia stem cells (LSC), and then targeting the markers or related signaling pathway have significantly improved the treatment response of chemotherapy in AML. Gemtuzumab targeting CD33 combined with intensive chemotherapy has been shown to increase the long‐term survival of core binding factor (CBF)‐AML patients from 50% to over 75%.^1^ Furthermore, midostaurin targeting Fms‐like tyrosine kinase (FLT3) mutation combined with chemotherapy has been recommended as frontline treatment in AML with FLT3 mutation AML by National Comprehensive Cancer Network (NCCN) guideline.^2^ Therefore, searching the special markers of leukemia, especially LSC, is the hit point of undergoing studies.

It is known that LSC is quiescent,^3^ and this property let the key point of leukemia resistance and relapse. Yet, how to label and eliminate LSC remains to be solved. Lapidot^4^ and Bonnet^5^ first described CD34^+^CD38^−^ AML as LSC since they could engraft severe combined immunodeficient (SCID) mice to produce colony‐forming progenitors. However, the normal hematopoietic stem cell (HSC) counterparts also express CD34,^6^ making it difficult to separate LSC from leukemic cells. Later, another study defined a new LSC marker interleukin‐3 receptor (CD123) as it is neglectable in normal HSC within the CD34^+^CD38^−^ compartment.^7^ With such marker, it is convenient to study the immunophenotypic and biological properties of LSC. Using the similar methods, Subsequent studies found several other LSC markers such as CD96,^8^ CD26,^9^ and CD25.^10^ Interestingly, all these markers are antigen molecules, indicating that they may also participate in the immune response in the bone marrow microenvironment. In order to apply the LSC markers to clinical practice, Ng et al established a 17‐gene LSC score (LSC17) to predicted the prognosis of AML patients.^11^

Our previous study in chronic myeloid leukemia (CML) found that Tim‐3 was highly expressed in a subset of CD34^+^CD38^+^CD123^high^ progenitors and could be a simple and less addressed surface marker of CML LSC.^12^ As a newly recognized LSC surface marker, Tim‐3 also plays a critical role in AML disease progression.^13^, ^14^ Two meta‐study analysis on Tim‐3 showed that upregulation of Tim‐3 was associated with poor prognosis in various of solid tumors,^15^, ^16^ but the clinical and biology features of Tim‐3 in AML remain to be determined. Herein our study found that upregulation of Tim‐3 could be used as a prognostic candidate predicting poor prognosis in AML patients. Furthermore, we found the biology of Tim‐3 was associated with immune response and LSC signaling, suggesting that Tim‐3 might be potential therapeutic target in AML.

## 2 MATERIALS AND METHODS

### 2.1 Data collection

The external AML cohorts covering transcriptional genes and clinical information were collected as follows. The Acute Myeloid Leukemia (TCGA, NEJM 2013) dataset and Acute Myeloid Leukemia (TCGA, Firehose Legacy) dataset were downloaded from cBioportal for Cancer Genomics (https://www.cbioportal.org/datasets).^17^, ^18^ Corresponding clinical characteristics, including gender, age, French‐American‐British classification (FAB classification), molecular risk, bone marrow transplantation information, white blood cell (WBC) count, FLT3 mutation, and event information were collected (Table 1). Three AML datasets (GSE37642‐GPL570, GSE71014, and GSE12417) from gene expression omnibus (GEO) database were downloaded and merged, correcting the batch effect with R package sva.^19^ We also collected 72 bone marrow specimens of newly diagnosed AML patients from March 2019 to December 2019 in the Department of Hematology, Nanfang Hospital, Southern Medical University as the internal cohort. All the patients were diagnosed with morphological assessment of the bone marrow in the identification of 20% or more myeloid blasts. Corresponding clinical characteristics, including age, gender, WBC count, FLT3 mutation, bone marrow transplantation information, molecular risk, OS time, and DFS time were collected (Table 2). Before treatment, we detected the Tim‐3 protein expression of CD45^+^CD34^+^ leukemia cells by flow cytometry. After that, the patients were treated with the 7 + 3 regimen of 7 days infused cytarabine (100‐200 mg/m2 daily) plus 3 days of an anthracycline (e.g., daunorubicin). We excluded the patients who were diagnosed and treated in other hospitals.

**TABLE 1:** Basic clinical information of 173 TCGA‐AML patients

	Tim3^high^ (%)	Tim3^low^ (%)	Total (%)	*p*
Number	147(84.97)	26(15.03)	173(100)	—
Sex				—
Male	79(45.66)	13(7.51)	92(53.18)	0.89
Female	68(39.31)	13(7.51)	81(46.82)	—
Age				—
>=60	67(38.73)	15(8.67)	82(47.40)	0.35
<60	80(46.24)	11(6.36)	91(52.60)	—
FAB				—
M3	10(5.78)	6(3.47)	16(9.25)	0.02
non_M3	137(79.19)	20(11.56)	157(90.75)	—
Molecular_risk				—
Good or intermediate	110(63.58)	15(8.67)	125(72.25)	0.16
Poor	35(20.23)	10(5.78)	45(26.01)	—
NA	2(1.16)	1(0.58)	3(1.73)	—
Bone marrow Transplantation				—
Yes	63(36.42)	10(5.78)	73(42.20)	0.84
No	84(48.55)	16(9.25)	100(57.80)	—
WBC (×10^9^)				—
>=100	15(8.67)	1 (0.58)	16(9.25)	0.47
<100	132 (76.30)	25 (14.45)	157(90.75)	—
FLT3 mutation				—
Yes	43(24.86)	7(4.05)	50(28.90)	0.99
No	98(56.65)	18(10.40)	116(67.05)	—
NA	6(3.47)	1(0.58)	7(4.05)	—
Event				—
Dead	103(59.54)	11(6.36)	114(65.90)	0.01
Alive	44(25.43)	15(8.67)	59(34.10)	—

Abbreviations: FAB M3, acute promyelocytic leukemia; FLT3, FMS‐like tyrosine kinase 3; NA, not applicable; TCGA‐AML, the Cancer Genome Atlas‐Acute myeloid leukemia; Tim‐3, T‐cell immunoglobulin mucin‐3; WBC, white blood cell.

**TABLE 2:** Information of 72 AML patients in Nanfang Hospital

Characteristics		Number	%
Age	≥60	14	19
	<60	58	81
Gender	Male	37	51
	Female	35	49
WBC			
	>=100		
	<100		
FLT3 mutation	Yes	20	28
	No	52	72
Bone marrow Transplantation	Yes	33	46
	No	39	54
Molecular risk	Good or Intermediate	37	51
	Poor	35	49
Tim‐3	Tim‐3^high^	32	44
	Tim‐3^low^	40	56

Abbreviations: FLT3, FMS‐like tyrosine kinase 3; Tim‐3, T‐cell immunoglobulin mucin‐3; WBC, white blood cell.

### 2.2 Flow cytometry

EDTA‐anticoagulated fresh bone marrow aspirates from 72 patients of our internal cohort were analyzed. Surface and intracellular antigen detection was performed on fresh bone marrow samples within 2 hours by multicolor flow cytometry. Leukemic stem and progenitor cells (LSC /LPC) were gated according to their CD45/CD34 properties. APC‐Cy7‐labeled anti‐CD45 (mouse monoclonal; clone2D1; #348795), PE‐Cy7‐labeled anti‐CD34 (mouse monoclonal; clone8G12; #348791), PE‐labeled anti‐Tim‐3 (mouse monoclonal; clone7D3; #563422), and isotype matched control antibodies (mouse IgG1 κ APC‐Cy7, #348792; mouse IgG1 κ PE‐Cy7, #348788; mouse IgG1 κ PE,#554680) were purchased from BD Biosciences (BD Biosciences, Franklin Lakes, NJ, USA). For surface antigen staining, 1 × 10^6^ cells were incubated for 30 min with 10 μl of appropriately diluted monoclonal antibody conjugates. The CD45, CD34, and Tim‐3 protein expression of AML specimens from the internal cohort were detected by FACSCantoII flow cytometer (BD Biosciences, Franklin Lakes, NJ, USA) with FACSuite (BD Biosciences) software, and the data were analyzed by FlowJo software (version 10.5.3).

### 2.3 Survival analysis

We divided patients into the Tim‐3^high^ and Tim‐3^low^ groups based on the Tim‐3 expression by X‐tile, a bio‐informatics tool for assessing the prognostic value of biomarkers.^20^ The Kaplan–Meier (K–M) curve was used to describe the overall survival (OS) and disease‐free survival (DSF) of the Tim‐3^high^ and Tim‐3^low^ groups,^21^ and then assessed the statistical significance between these two groups by log‐rank test.^22^ The multivariate Cox regression analyses were conducted to investigate the prognostic value of Tim‐3 expression.

### 2.4 Statistical analyses

All the statistical analyses were conducted using R software (version 4.1.1) unless otherwise stated. All statistical results with a *p* < 0.05 were considered significant. Associations between clinical factors in group comparisons were performed by the Mann–Whitney nonparametric *U*‐test and Kruskal–Wallis test or Fisher's exact tests. For the survival analysis, the Kaplan–Meier survival curves and the log‐rank test were used. The OS was calculated from the diagnosis to the last observation or death. Complete remission (CR) was defined as <5% blasts in bone marrow aspirates, peripheral blood lacking leukemia blasts, and restoration of peripheral blood counts. The DFS was measured from CR date to relapse or last follow‐up. DFS and OS were analyzed by the Kaplan–Meier method. Multivariate analyses on categorized data were performed using Cox proportional hazards mode, which were fitted to evaluate the effects of patient characteristics on DFS and OS.

### 2.5 Identification of differentially expressed genes

In TCGA‐AML cohort, we identified the differentially expressed genes (DEGs) between the Tim‐3^high^ and Tim‐3^low^ groups by R package limma. False discovery rate (FDR) was used to correct the statistical significance of the multiple test. The mRNAs with |log2(fold change (FC))| > 0.5 and FDR <0.05 were regarded as differentially expressed.

### 2.6 GO, KEGG, GSEA, and immune infiltration analyses

In TCGA‐AML cohort, between the Tim‐3^high^ and Tim‐3^low^ groups, gene ontology (GO) terms, Kyoto Encyclopedia of Genes and Genomes (KEGG) pathway enrichment and Gene set enrichment analyses (GSEA) were annotated to identify if Tim‐3 expression is associates with the LSC signature.^23^, ^24^, ^25^ Immune and stromal scores, as well as the estimation of stromal and immune cells in malignant tumor tissues using expression data (ESTIMATE) were also calculated using the ESTIMATE algorithm (https://sourceforge.net/projects/estimateproject/) in order to compare the immune infiltration between the Tim‐3^high^ and Tim‐3^low^ groups.

## 3 RESULTS

### 3.1 Differential expression of Tim‐3 between AML patients and healthy individuals

**FIGURE 1:**
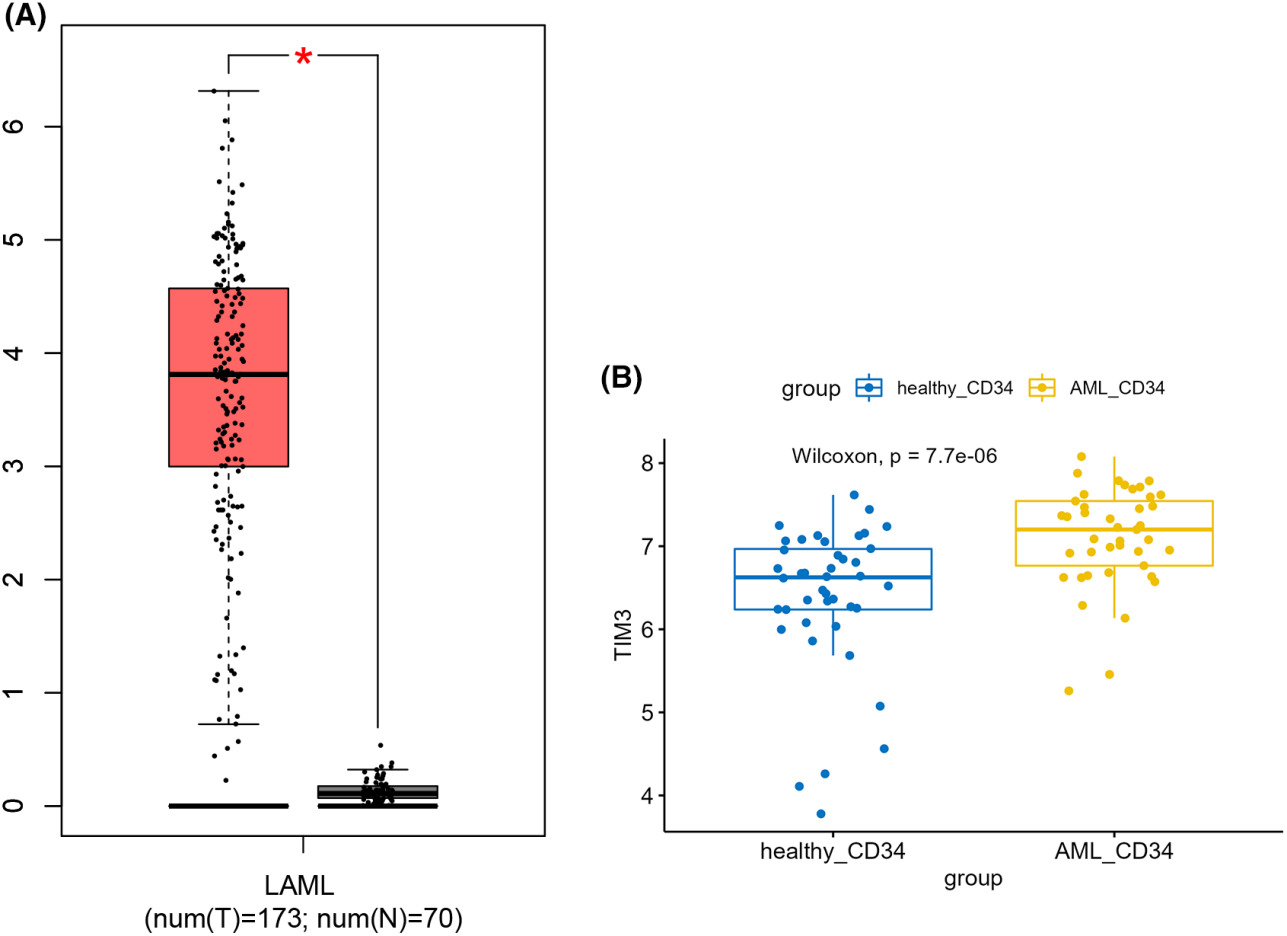
Comparison of Tim‐3 expression between AML and healthy donors. Compare mononuclear cells of AML patients from TCGA‐AML cohort with healthy individuals from GTEx dataset (A), compare LSC/LPC with normal HSPC from GSE63270 cohort in GEO database (B). AML, Acute myeloid leukemia; GEO, gene expression omnibus; GTEx, genotyping tissue expression; TCGA, the cancer genome atlas; Tim‐3, T‐cell immunoglobulin mucin‐3.

Gene expression profiling interactive analysis (GEPIA) is a web server that records RNA‐sequencing (RNA‐seq) information based on TCGA and genotyping tissue expression (GTEx).^26^ By this web, we compared the Tim‐3 expression between AML patients from TCGA dataset (*n* = 173) and healthy donors from GTEx dataset (*n* = 70). It showed that Tim‐3 was significantly higher in AML patients than healthy individuals (Figure 1A). This is consistent with the results of Amany et al.^27^ As previously mentioned, Tim‐3 is associated with LSC /LPC, so we also compare the expression of Tim‐3 between LSC /LPC and normal hematopoietic stem progenitor cell (HSPC) of GSE63270 cohort. It showed that the Tim‐3 gene expression is also upregulated in LSC/LPC (Figure 1B), this result was consistent with Kikushige's findings.^13^ Since Tim‐3 gene is markedly elevated in leukemic blast and LSC/LPC, it may mediate the origin and progression of leukemia.

### 3.2 Comparison of survival between the Tim‐3^high^ group and Tim‐3^low^ group of TCGA‐AML cohort

**FIGURE 2:**
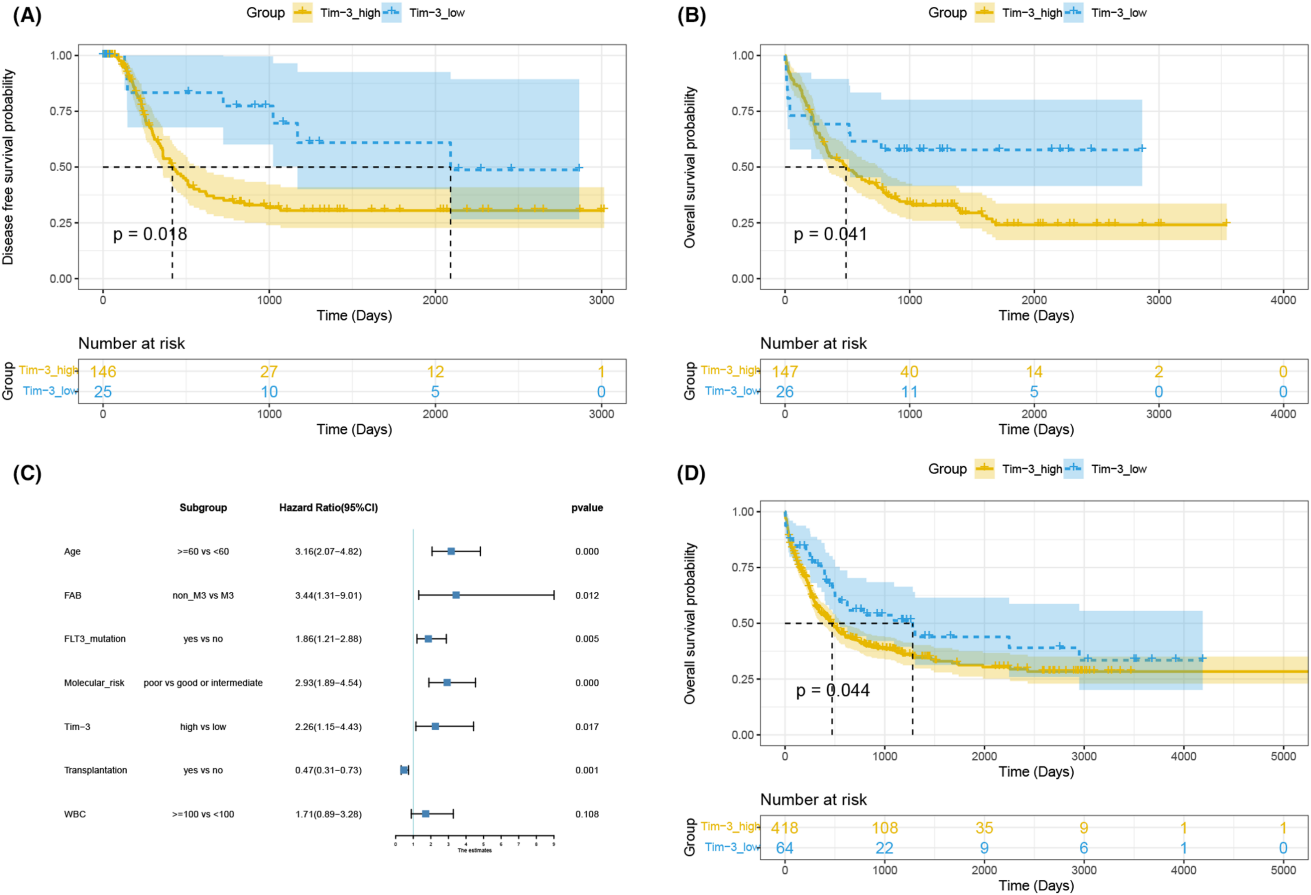
Comparison of survival between the Tim‐3^high^ group and Tim‐3^low^ group. The DFS difference of TCGA‐AML cohort (A), the OS difference of TCGA‐AML cohort (B), the cox regression analysis of TCGA‐AML cohort (C), the OS difference of GEO‐AML cohort (D). DFS, disease free survival; GEO, gene expression omnibus; OS, overall survival; TCGA, the cancer genome atlas; Tim‐3, T‐cell immunoglobulin mucin‐3.

In order to explore whether the Tim‐3 expression has a prognosis value in AML, we download 173 TCGA‐AML patients from the cBioportal database by selecting the Acute Myeloid Leukemia (TCGA, NEJM 2013) dataset. In this cohort, there were 92 male patients, 81 female patients, 81 patients over 60 years of age, 126 patients with good or moderate molecular risk stratification, 47 patients with poor prognosis, 73 patients that underwent bone marrow transplantation, and 100 that did not. Of these 173 patients, 114 have died and 59 survived. Since the Tim‐3 expression is continuous variable data, we transformed it into a binary variable in which the cut‐off value was determined by the X‐tile software. The optimal cut‐off value divided TCGA‐AML patients into the Tim‐3^high^(*n* = 147) and Tim‐3^low^(*n* = 26) groups (Table S1). The basic clinical information of TCGA‐AML patients are shown in Table 1. The survival and survminer packages were used to compare the OS and DFS of these two groups. The results showed that the OS and DFS of patients in the Tim‐3^high^ group were worse than those in the Tim‐3^low^ group (Figure 2A, B), suggesting that Tim‐3 expression has a negative correlation with patient prognosis. Consistent with the conclusion that Tim‐3 is used as a marker of LSC, the higher expression of Tim‐3, the worse the prognosis is. This may be related to the insensitivity of Tim‐3^+^ cells to chemotherapy, or the proliferation of residual Tim‐3^+^ cells after chemotherapy. In conclusion, Tim‐3, as a marker of LSC, may mediate the origin and recurrence of AML, leading to a poor prognosis.

Although the univariate analysis suggested that Tim‐3 expression in leukemia cells was associated with poor prognosis, it was affected by age, molecular mutations, and bone marrow transplantation, etc. Therefore, we performed a multivariate cox regression analysis to correct for these factors. The age, molecular risk stratification, leukocytes number at the initial diagnosis, and the expression of Tim‐3 were included in this analysis. The results showed that after adjusting for factors such as age and transplantation, Tim‐3 still had predictive significance for patient prognosis (Figure 2C). This indicates that leukocyte Tim‐3 was an independent prognostic factor for AML. To further validate this conclusion, we performed a survival analysis using the GEO dataset. The GSE37642‐GPL570, GSE71014, and GSE12417 datasets were downloaded using the GEOquery package and merged these cohorts using the sva package to remove the batch effect. We also divided these patients into two groups based on Tim‐3 expression and the grouping information seen in Table S2. The survival analysis also showed that the higher the expression of Tim‐3, the worse the OS of patients (Figure 2D).

### 3.3 Different expression genes analysis and functional enrichment analysis based on Tim‐3 gene expression

Most of the previous studies about Tim‐3 have focused on its role in LSC, but there are no reports evaluating the difference biological feature between Tim‐3^high^ and Tim‐3^low^ AML patients. Since patients with higher Tim‐3 expression had worse prognosis, we hypothesized that AML patients in the Tim‐3^high^ group might have more stemness gene upregulation than those in the Tim‐3^low^ group.

**FIGURE 3:**
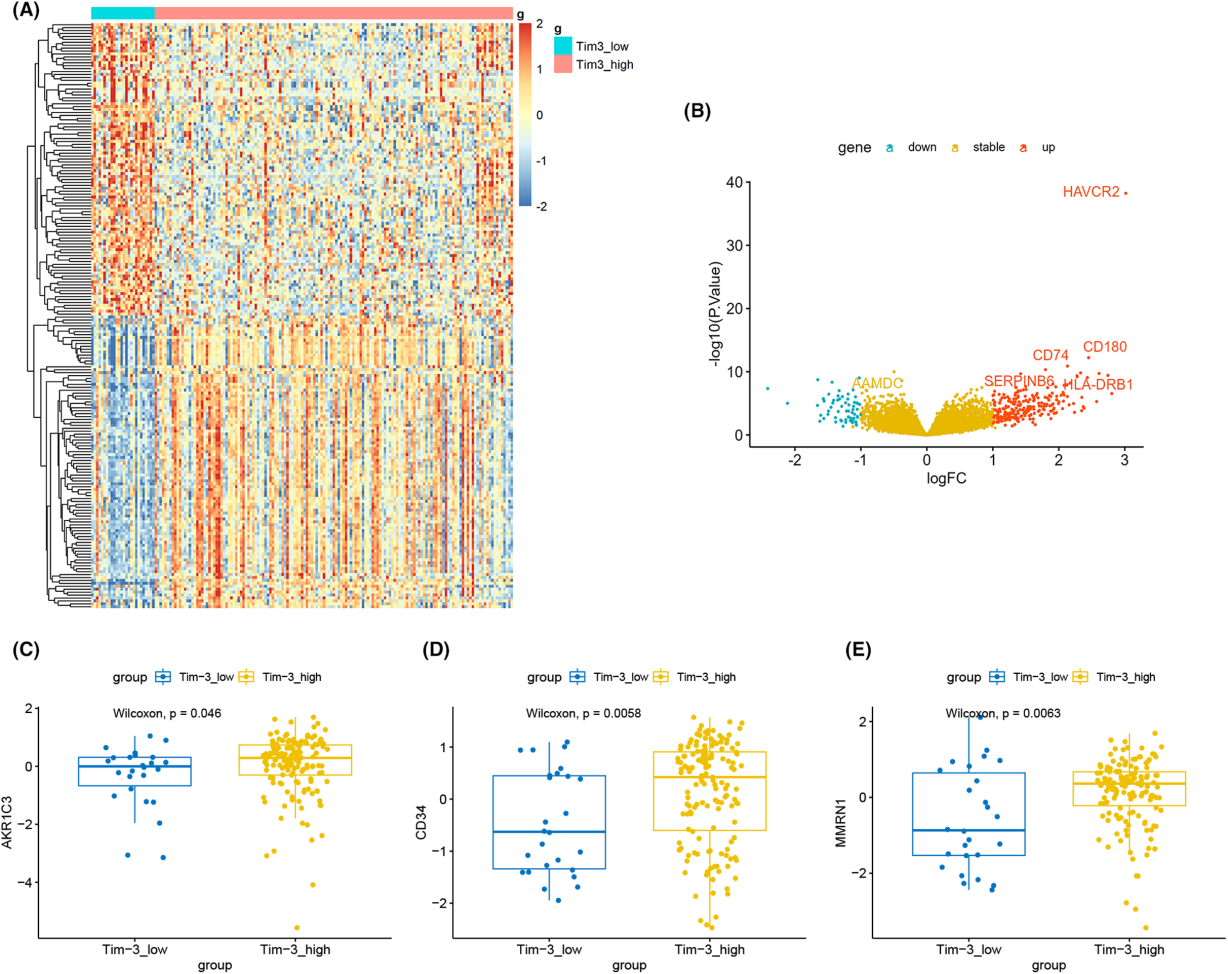
Comparison of gene expression between the Tim‐3^high^ group and Tim‐3^low^ group of TCGA‐AML. The heatmap (A) and volcano (B). There stemness associated genes were upregulated in the Tim‐3^high^ group (C, D, E).

Therefore, we choose the Acute Myeloid Leukemia (TCGA, Firehose Legacy) dataset in the format of reads per kilobase per million mapped reads (RPKM) to identify the differentially expressed genes. With the screening criteria described above, we found a total of 297 genes in the Tim‐3^high^ versus Tim‐3^low^ groups were different. Of these different expression genes (DEGs), there are 63 downregulated genes and 234 upregulated genes (Figure 3A, B). The detailed information was shown in Table S3. Stanley et al. proposed in a study in 2016 that using 17 stemness genes to build an AML prediction model which is of great significance for predicting patient prognosis.^11^ With the Acute Myeloid Leukemia (TCGA, NEJM 2013) dataset (data_RNA_Seq_v2_mRNA_median_all_sample_Zscores.txt), we found that among those 16 stemness genes (one gene named SMIM24 wasn't tested in this cohort), *AKR1C3* (*p* = 0.046), *CD34* (*P* = 0.0058), and *MMRN1* (*P* = 0.0063) were highly expressed in the Tim‐3^high^ group (Figure 3C‐E) (the data were normalized with R package limma), and the rest genes were not significant difference(*p* > 0.05) (Figure S1). Notably, CD34 is a well‐known marker of stem/progenitor cells and represents cells with self‐renewal ability. These data strongly indicate that Tim‐3 has a positive correlation with stemness genes.

**FIGURE 4:**
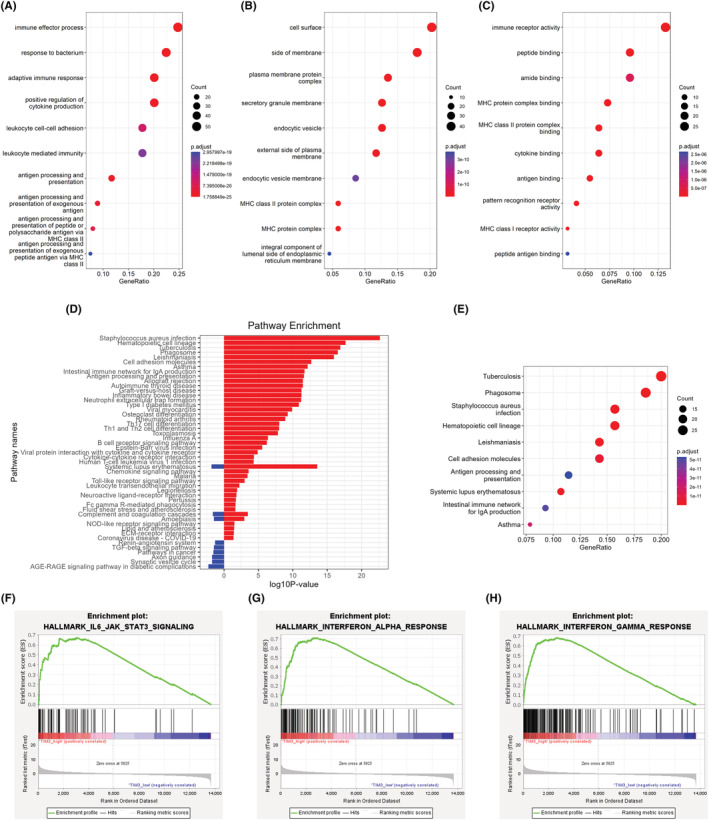
Functional enrichment analysis. Enrichment analysis of top 10 GO terms of biological process, cellular component and molecular function that contains the up genes in Tim‐3^high^ cohort (A, B, C). KEGG pathway analysis were performed as pathway annotation (D), and bubble chart of top 10 pathways enrichment by analyzing the up genes in Tim‐3^high^ cohort (E). GSEA analysis was performed to further screen the significant pathway between the Tim‐3^high^ group and the Tim‐3^low^ group (F, G, H). GO, gene ontology; GSEA, gene set enrichment analysis; KEGG, Kyoto Encyclopedia of Genes and Genomes.

Subsequently, the GO enrichment and KEGG analysis were performed to understand the differences in cell signaling pathways between the two groups. The GO results indicated that the genes upregulated in the Tim‐3^high^ group were associated with immune effector process, secretory granule membrane, and immune receptor activity, etc. (Figure 4A, B, C). In the KEGG enrichment analysis, we found pathways associated with immunity, cell adhesion molecules, and AML (Figure 4D). The top ten pathway enrichment analysis was shown in Figure 4E where bubble size represented gene number and color represented adjusted *p* value. Since Tim‐3 is not only expressed on LSC, but also expressed on immune cells, such as T cells, dendritic cells, and NK cells.^28^, ^29^ These results suggest that Tim‐3 is closely related to stem cell self‐renewal, as well as to immune response which is related to AML.

Next, we further screen the significant pathway between the Tim‐3^high^ group and Tim‐3^low^ group and found that there are nine pathways are significant different. The most significant pathways are associated with IL6‐JAK–STAT3‐signaling (Figure 4F), Interferon‐ alpha‐response (Figure 4G), and Interferon‐gamma‐response (Figure 4H). The more information about the whole pathways were shown in Table S4. Additionally, we also performed the GO enrichment analysis, KEGG analysis, and GSEA analysis based on the Tim‐3 expression in the GEO‐AML dataset, and the results are basically consistent with those of TCGA‐AML dataset (Figure S2).

### 3.4 Differences in immune infiltration between the Tim‐3^high^ and Tim‐3^low^ groups

**FIGURE 5:**
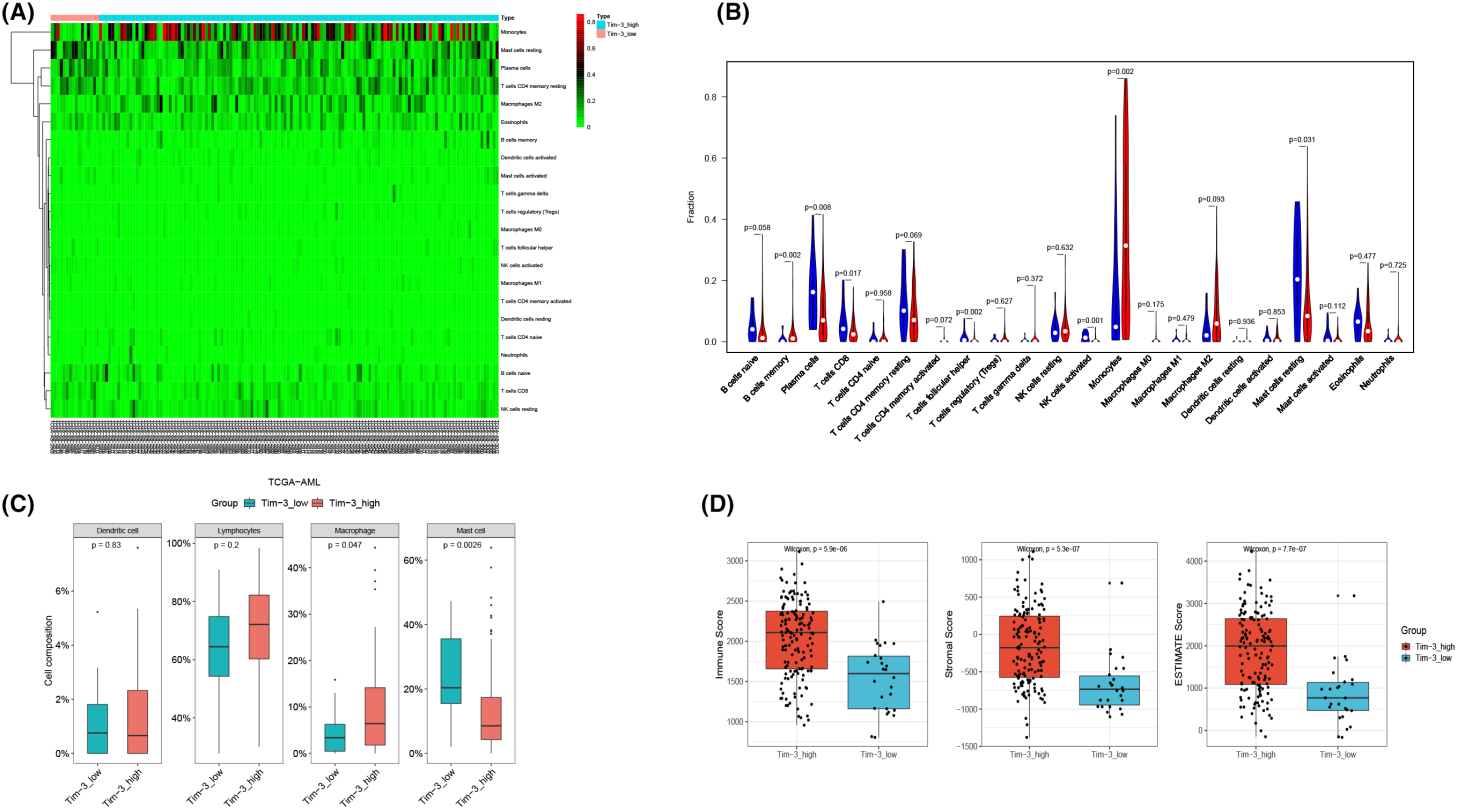
Immune and stromal scores between Tim‐3^high^ and Tim‐3^low^ are different. The different gene expression of 22 types of immune cells between Tim‐3^high^ and Tim‐3^low^: heatmap (A) and vioplot (B). 22 types of immune cells divide into four classes and different gene expression of these four classes between Tim‐3^high^ and Tim‐3^low^ (C). The immune score, stromal score, and Estimate score between Tim‐3^high^ and Tim‐3^low^ (D).

In addition to leukemia cells, the bone marrow microenvironment also includes macrophages, lymphocytes, monocytes, and myeloid‐derived suppressor cells (MDSC).^30^ Some immune cells play anti‐tumor effects, while others promote tumor progression. The above results suggest that AML patients with higher expression of Tim‐3 have a worse prognosis. Therefore, it is necessary to further understand whether there is a significant difference in immune infiltration between the Tim‐3^high^ and Tim‐3^low^ groups. We calculated the proportion of 22 types of immune cells in each sample based on bulk RNA‐seq data by Cibersort software. The results showed that the proportion of monocytes as well as macrophages were higher in the Tim‐3^high^ group (Figure 5A, B). As we know that tumors in the microenvironment could recruit monocytes through chemokines, and when monocytes differentiated into M2 macrophages, they could inhibit the immune response, thereby promoting tumor progression.^31^ In this study, M2 macrophages in the Tim‐3^high^ group showed a higher trend than in the Tim‐3^low^ group, suggesting that the Tim‐3^high^ group may exist immunosuppression. Previous research showed that 22 types of cells can be divided into four categories, namely dendritic cells, lymphocytes, macrophages, and mast cells.^32^ The analysis found that the overall proportion of macrophages in the Tim‐3^high^ group was higher, and the differences were statistically significant. While total lymphocytes did not differ between the two groups, CD4^+^ T cells had a higher proportion in the Tim‐3^low^ group, and CD8^+^ T cells also tended to be higher, suggesting that the Tim‐3^low^ group exhibited a stronger immune effect. These results were shown in Figure 5C.

The ESTIMATE software can calculate the total immune score and stromal score according to the proportion of 22 kinds of immune cells, and these two scores represented the proportion of immune cells and mesenchymal cells, respectively. Some researchers have explored the correlation between immune infiltration and prognosis in the TCGA‐AML cohort and confirmed that patients with a high immune score had a worse prognosis than those with a low immune score. This difference was statistically significant, and patients with a high stromal score also showed a worse prognosis than the lower group, although this was not statistically significant.^33^, ^34^ In this study, the ESTIMATE calculation showed that the immune, stromal, and ESTIMATE scores of the Tim‐3^high^ group were significantly higher than those of the Tim‐3^low^ group, which further verified that the Tim‐3^high^ group had a worse prognosis considering the aspect of immune infiltration (Figure 5D).

### 3.5 The relationship between Tim‐3 protein expression of LSC/LPC and AML prognosis

The above data showed that the expression of stemness markers in the Tim‐3^high^ group is higher, suggesting that Tim‐3 positive tumor cells are closely related to leukemia stem cells. Previous studies have suggested that higher gene expression of Tim‐3 in bone marrow cells indicates a worse prognosis in AML patients,^27^ and the external cohort analyzed above also supports this conclusion. However, there is no relevant research on the relationship between LSC/LPC Tim‐3 protein expression and patient prognosis. Since Tim‐3 is involved in cellular stem cell signaling, we hypothesized that Tim‐3 expression in stem progenitor cells may also be associated with AML progress. Additionally, Tim‐3 expression in LSC/LPC cells may be more closely related to patient prognosis than Tim‐3 expression in bulk cells and is more accurate for predicting patient prognosis.

**FIGURE 6:**
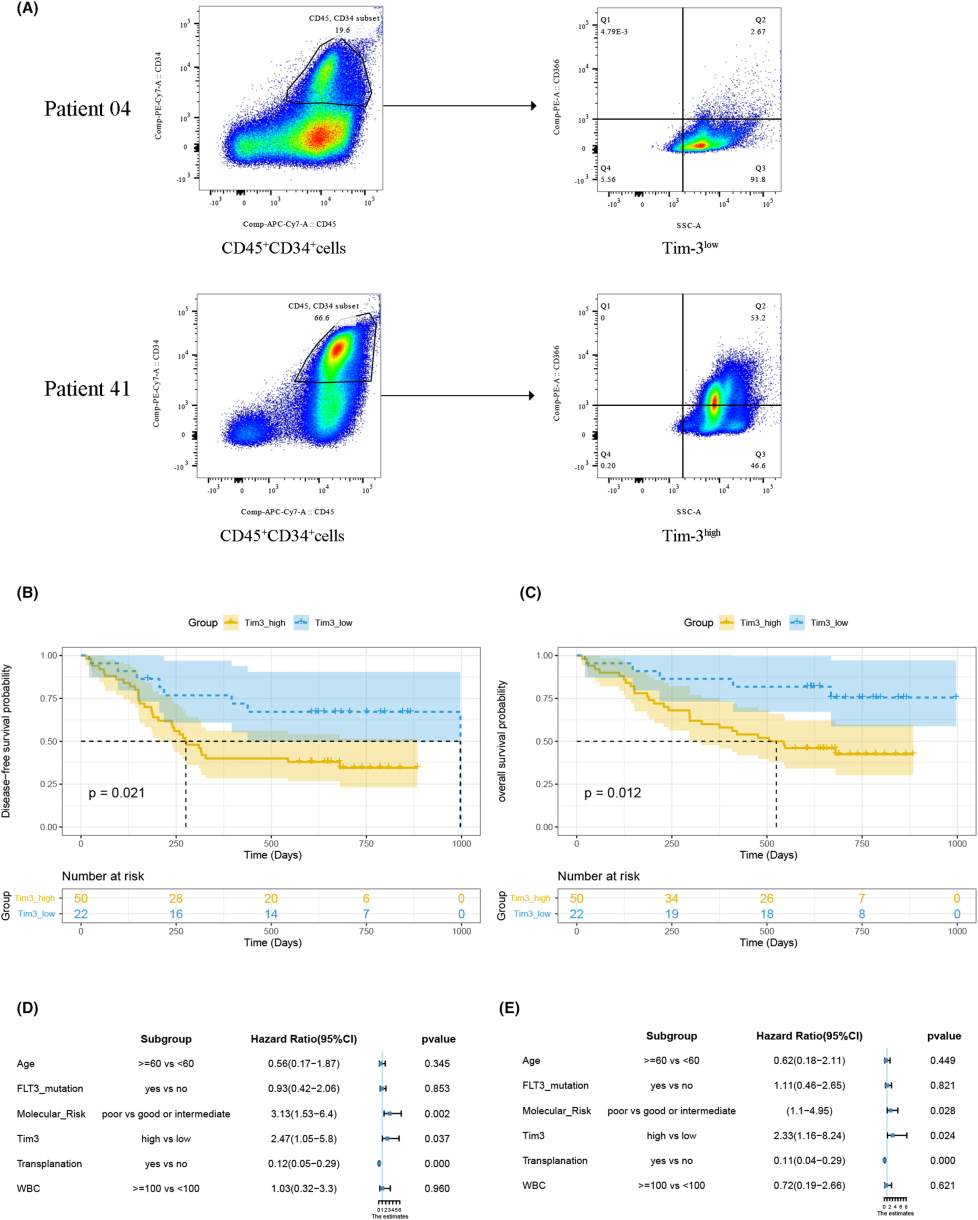
Comparison of survival between the Tim‐3^high^ group and the Tim‐3^low^ group of internal cohort. The gate strategy for flow cytometry data to divided patients into two groups based on Tim‐3 expression (A). The DFS difference (B) and the cox regression analysis (D), the OS difference (C) and the cox regression analysis (E). DFS, disease free survival; OS, overall survival; Tim‐3, T‐cell immunoglobulin mucin‐3.

Based on this assumption, we collected bone marrow cells of newly diagnosed AML patients who were hospitalized in the Department of Hematology, Nanfang Hospital from March 2019 to December 2019, and detected the expression of Tim‐3 of CD45^+^CD34^+^ stem/progenitor cells by flow cytometry. To verify the above results, and explore whether stem/progenitor Tim‐3 can also predict the prognosis of patients, a total of 72 patients were enrolled in this study, including 37 men and 35 women, most of them were young and middle‐aged patients. There were five patients over 60 years old, accounting for 7%, 20 patients with a FLT3 gene mutation, and 33 patients underwent transplantation. There were 37 cases with good and moderate prognosis, and 35 cases with poor prognosis according to molecular risk. In the same way as the previous method, the X‐tile software was used to find the optimal cut value according to the positive rate of Tim‐3 on CD34^+^ cells of AML patients, and the patients were divided into the Tim‐3^high^ and Tim‐3^low^ groups (Figure 6A). The basic clinical information of the patients is shown in Table 2. And the Tim‐3 grouping information is seen in the Table S5.

The OS and DFS of the two groups of patients were compared using the survminer and survival packages. The results showed that the AML patients in the Tim‐3^high^ group had poor DFS and OS than those in the Tim‐3^low^ group, further confirming that Tim‐3 is of great significance for predicting the prognosis of AML patients (Figure 6B, C).

To correct for the influence of other factors on Tim‐3, a multivariate cox regression analysis was performed. Multiple factors that have been well established to influence AML prognosis were incorporated into the model, including age, WBC count, FLT3 mutation, and molecular genetic risk stratification. Regardless of EFS or OS, high Tim‐3 is an independent risk factor (Figure 6D, E), which is consistent with the results of the TCGA‐AML and GEO datasets described above, suggesting that Tim‐3 of CD34^+^ leukemia cells could be a potential therapeutic target.

## 4 DISCUSSION

Persistence of LSC is an important cause of relapse/refractory AML, and finally lead to treatment failure.^35^, ^36^ Previous study identified multiple pathways that distinguish LSC from HSC, such as adherens junction, regulation of the actin cytoskeleton, apoptosis, and wingless/integrated (WNT) signaling.^37^ Recently, numbers of targeted therapy and immunotherapy studies for LSC are ongoing.^38^, ^39^ In this study, we have made two important findings. First, evidence supporting the prognostic value of Tim‐3 gene in leukemic cells has been well defined.^27^ However, for the first time, we revealed that upregulation of Tim‐3 gene in the LSC was also associated with poor prognosis in AML. Meanwhile, we confirmed that Tim‐3 protein was also over expressed in LSC/LPC and that made poor prognosis in AML by our internal cohort. Second, we revealed that Tim‐3 not only maintained the self‐renew function of LSC which was consistent with other findings, but also promoted immunosuppression and immune escape in AML. Our findings suggested that Tim‐3 might be potential therapeutic target of LSC in AML.

Tim‐3 is originally discovered as a surface marker of T cells^40^ as an immune checkpoint and T cell exhaustion.^41^ Kikushige and colleagues found that Tim‐3 was highly expressed in AML stem progenitor cells, but not in normal HSCs, demonstrating that Tim‐3 could be used as a marker of LSCs.^13^ Subsequent study revealed that Tim‐3 activated the nuclear factor (NF)‐κB pathway and the WNT pathway after binding to the ligand galectin (Gal)‐9, thereby maintaining the self‐renewal of leukemia cells.^14^ In addition, the expression of pro‐apoptotic proteins BCL‐associated X (BAX) and SIVA is reduced after co‐culture of Tim‐3^+^ AML cells with Gal‐9 or high mobility group box 1(HMGB1).^42^ In addition to AML, we previously found that leukemic progenitor cells in CML also highly expressed Tim‐3 and β‐catenin, suggesting a correlation between the Tim‐3 and WNT pathways.^12^ These studies suggest that Tim‐3 involves in the proliferation of LSC and might mediate the progression and drug resistance of AML.

Tim‐3 has been proved as a prognostic marker in a variety of solid tumors, and its expression level is negatively correlated with prognosis.^43^, ^44^, ^45^ Although Tim‐3 serves as a marker of LSC, its prognostic value in LSCs of AML remains unclear. The present study demonstrated that upregulation of Tim‐3 in LSCs was associated with poor prognosis in AML. We further explored the biology of Tim‐3 and found that the IL6‐JAK–STAT3 and IL2‐STAT5 signaling were enriched in the Tim‐3^high^ group. In accordance, one study showed that multiple pathways of KEGG were enriched in LSC, especially adherens junction, T‐cell receptor signaling and JAK‐STAT signaling pathways.^37^ In addition, Peron's study on auxanology found that JAK‐STAT3 signaling was essential for maintaining cell stemness by Wnt/β‐catenin pathway.^46^ Another research showed that Stat3 could promote pro‐oncogenic inflammatory pathways like nuclear factor‐kB(NF‐kB).^47^ These studies illustrate that Tim‐3 may activate LSC signaling via Stat3. Furthermore, in consistent with previous studies,^33^ we explored immune infiltration and found that the Tim‐3^high^ group patients had higher levels of immune infiltration, especially in monocytes, macrophages, and T cells. Since AML cells could attenuate anti‐tumor ability of immune cells,^48^ and high expression of immune checkpoint in the bone marrow leukemia cells correlated with poor prognosis,^49^ and our GO enrichment analysis of bulk RNA‐seq showed that upregulated genes in the Tim‐3^high^ group were enriched in immune response and cytokine binding et al., we speculate that Tim‐3 has a dual role in AML (a): Tim‐3 expressed in LSC promotes leukemogenesis. (b) Tim‐3 expressed in immune cells leads to immune dysregulation. Therefore, in addition to mediating LSC‐related signaling pathways, Tim‐3 may also affect the efficacy of AML treatment through immune responses.

In line with our data that Tim‐3 overexpresses in LSC/LPC and poorly impacts on the prognosis of AML, Tim‐3 has been shown to be a promising target in the treatment of AML. Sabatolimab (MBG453) is a Tim‐3 monoclonal antibody that blocks the interaction between Tim‐3 and its ligands. Preclinical study showed that MBG453 promoted LSC apoptosis, and enhanced the anti‐leukemia effect of immune cells by attenuated the pro‐leukemic bone marrow microenvironment. Now, a Phase I/II clinical trial of MBG453 single drug used in the treatment of high‐risk MDS as well as relapsed/refractory AML is ongoing and shows encouraging efficacy and good safety.^50^, ^51^

Taken together, our study showed that Tim‐3 was an independent risk factor at the level of leukemic cells and LSCs in AML patients, and high expression of Tim‐3 in leukemic blast represented LSC signaling and suppression of immune response. Furthermore, considering the critical dual role of Tim‐3 in LSC and immune checkpoint, targeting Tim‐3 not only eliminates LSC, but also balances the immune system, as one stone two birds. There are numerous ongoing clinical trials of Tim‐3 inhibitors or Tim‐3 monoclonal antibodies for the treatment of AML and MDS. However, defining the high and low expression of Tim‐3 required further elucidation in larger cohort study. It is necessary to conduct multi‐center clinical studies including more patients to clarify the threshold for grouping Tim‐3 expression levels as well as evaluating the benefit of Tim‐3‐targeting therapy. Additionally, this study confirmed the biology function of Tim‐3 mainly by bioinformatics analysis which is limited in terms of accuracy. So, it's necessary to perform in vitro experiments with overexpression and knockdown of Tim‐3 for further validation.

## 5 CONCLUSION

In summary, through comprehensive analysis of multi‐center and multi‐omics AML patients' data, we found for the first time (a): Tim‐3 at the level of gene and protein expression in AML LSCs is closely related to prognosis; (b) The high expression of Tim‐3 in LSC is closely related to stemness genes, self‐renewal, immune infiltration, and immune escape. This study increases our understanding of the prognosis value and biological feature of Tim‐3 and provide more evidence for targeting Tim‐3 therapies ongoing.

## AUTHOR CONTRIBUTIONS

**Zhengwei Wu:** Data curation (lead); formal analysis (lead); investigation (lead); writing – original draft (lead). **Jiawang Ou:** Data curation (supporting). **Nannan Liu:** Methodology (equal). **Zhixiang Wang:** Data curation (equal). **Junjie Chen:** Writing – review and editing (equal). **Zihong Cai:** Supervision (equal). **xiaoli Liu:** Writing – review and editing (equal). **Xiao Yu:** Conceptualization (equal). **Min Dai:** Writing – review and editing (equal). **Hongsheng Zhou:** Conceptualization (lead); funding acquisition (lead); project administration (lead); resources (lead); supervision (lead); writing – review and editing (equal).

## FUNDING INFORMATION

This study was supported by National Natural Science Foundation of China (NSFC 82170163, 81970147, 81770170, to HSZ), the Science and Technology Planning Project of Guangdong Province (No. 2017A030313601 to HSZ).

## CONFLICT OF INTEREST

All authors declare no conflict of interest.

## ETHICS STATEMENT

This study was performed according to the Declaration of Helsinki principles and approved by the Ethics Committee of Affiliated Nanfang Hospital of Southern Medical University. All participants provided written informed consent.

## Supporting information


Figure S1.

Figure S2.

Table S1.

Table S2.

Table S3.

Table S4.

Table S5.

## Data Availability

The TCGA‐AML cohort analyzed in this study can be found in the cBioPortal for Cancer Genomics (https://www.cbioportal.org/datasets) and the GEO‐AML cohorts can be found in GEO (https://www.ncbi.nlm.nih.gov/geo/query/acc.cgi?acc=GSE37642, https://www.ncbi.nlm.nih.gov/geo/query/acc.cgi?acc=GSE71014 and https://www.ncbi.nlm.nih.gov/geo/query/acc.cgi?acc=GSE12417).
