# Supplementary figures and images for: Upregulation of Tim‐3 is associated with poor prognosis in acute myeloid leukemia
Source: Cancer Med. 2022 Dec 21;12(7):8956–69. doi: 10.1002/cam4.5549 (PMC10134367; doi:10.1002/cam4.5549)

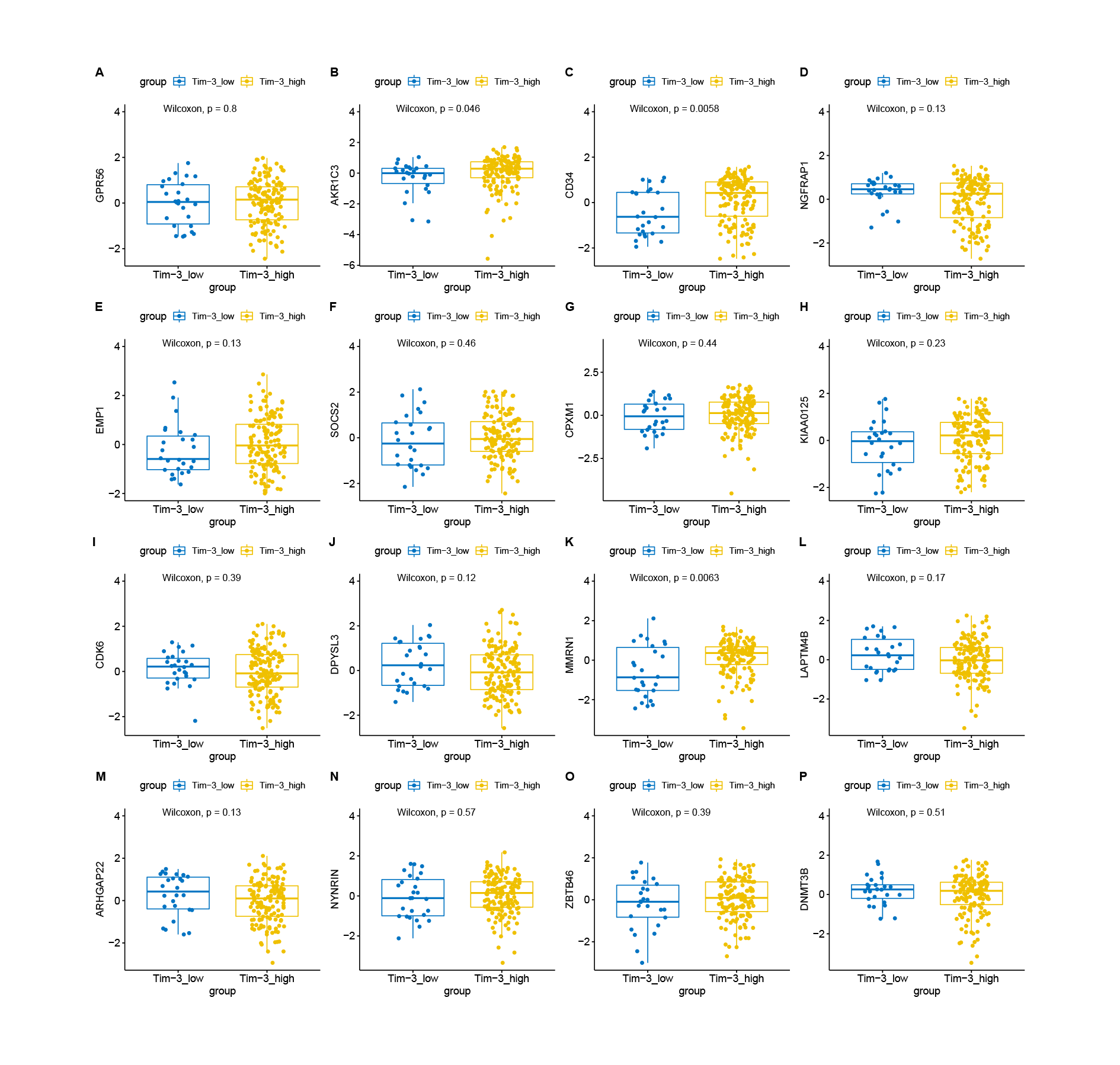

Supplement: Supplementary file 1 — Figure S1. [file CAM4-12-8956-s003.tif]

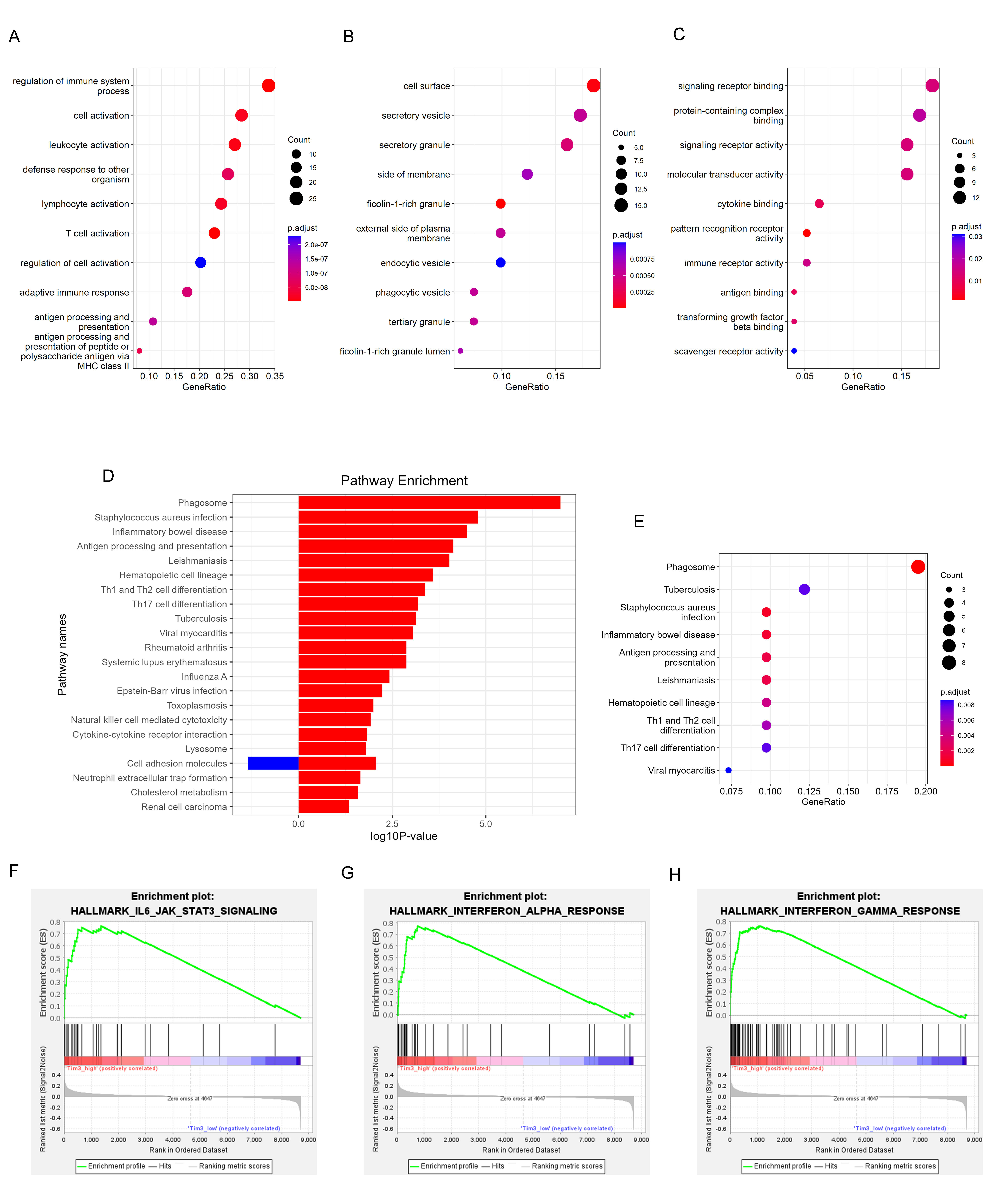

Supplement: Supplementary file 2 — Figure S2. [file CAM4-12-8956-s006.tif]
